# Launching the Latin American Epidemiological Cooperation relating to Noncommunicable Diseases

**DOI:** 10.1590/1516-3180.2018.1363070518

**Published:** 2018-03-29

**Authors:** Paulo Andrade Lotufo

**Affiliations:** I MD, DrPH. Full Professor, Department of Internal Medicine, Faculdade de Medicina da Universidade de São Paulo (FMUSP), São Paulo (SP), Brazil.

Latin America and the Caribbean form one of the world’s macroregions and comprise 50 independent countries and a few colonies, with a total of approximately 650 million inhabitants. Although these countries have almost twice the population of the United States, the combined gross national product of Latin American and Caribbean countries is only 55% of that of the United States.^1^ The socioeconomic differences between Latin American and Caribbean countries and the United States are reflected in educational, health-related and scientific indexes that show Latin American and Caribbean countries unfavorably. The amount of money spent on medical care and, especially, to support medical and health-related research in the United States is immensely greater than in Latin American and Caribbean countries. In contrast, the principle of universal health care coverage that has been adopted in most Latin American and Caribbean countries faces stiff opposition in the United States.^2^

Despite these differences, the epidemiological profile of all countries in the Americas is much closer than what was seen half a century ago. Infectious and parasitic diseases in Latin America have declined with only a few exceptions, such as arbovirus-borne diseases. Likewise, disorders relating to undernutrition and maternal care have faded away in most Latin American and Caribbean countries. Noncommunicable diseases such as cardiovascular, respiratory, renal, mental and osteoarticular diseases have become the most important cause of death, morbidity and disability on both sides of the border between the United States and Latin American and Caribbean countries.^3^ One example of this is the astonishingly temporal trends of obesity prevalence observed between Latin American and Caribbean countries and the United States.^4^ The epidemiological profile and priorities in Latin American and Caribbean countries were discussed previously on this page, in an analysis on Argentina, Brazil, Colombia and Mexico, using data from the Global Burden of Diseases study. Briefly, the priorities for noncommunicable diseases have been determined to be: (1) control of alcohol intake; (2) detection, treatment and control of hypertension; (3) prevention of stroke and coronary heart disease secondary care; (4) prevention of obesity and diabetes; and (5) screening for chronic kidney disease.^5^

To face the challenge of noncommunicable diseases, strong scientific support from epidemiological studies has been necessary. Despite the enormous contribution of the United States towards the epidemiology of chronic diseases, such as through the seminal Framingham Heart Study,^6^ and the contributions made by other countries, the realities of Latin American and Caribbean countries impose a specific approach. Specific longitudinal studies addressing particular issues relating to ethnicity, diet and socioeconomic determinants are needed. Fortunately, the challenge of understanding the determinants of chronic diseases in Latin American and Caribbean countries has indisputably been addressed by local scientists and public health authorities in Argentina,^7^ Brazil,^8^ Chile,^7^^,^^9^ Mexico,^10^ Peru^11^ and Uruguay.^7^

In April 2018, it was possible to organize an initial summit event in Chile, which was called the “First Meeting on Latin American Population-Based Cohorts for Studies on Chronic Diseases” (I Jornada de Cohortes Poblacionales Latinoamericana para el Estudio de Enfermedades Crónicas, COPLAS). This meeting brought together researchers from 12 studies. Table 1 describes the five ongoing cohort studies in Latin American and Caribbean countries among adults without any specific disease.^7^^,^^8^^,^^9^^,^^10^^,^^11^

**Table 1:** Description of five ongoing Latin American cohorts among adults, addressing cardiovascular diseases (CVDs), cancer, diabetes and chronic pulmonary obstructive disease (COPD)

Acronym (reference)	Name	Country	Main aim	Number of participants	Start date	Website
CESCAS^7^	Center for Excellence in Cardiovascular Health in South America	Argentina, Chile and Uruguay	Prevalence, incidence and distribution of risk factors for CVDs, COPD and cancer	7,524 adults in 4 cities in 3 countries aged 35 to 74 years Random sampling	2010	https://estudiocescas.iecs.org.ar/
ELSA-Brasil^8^	Brazilian Longitudinal Study of Adult Health	Brazil	Development and progression of clinical and subclinical chronic diseases, particularly CVDs and diabetes.	15,105 civil servants aged 35-74 years in 6 cities in Brazil	2008	http://www.elsa.org.br/
MAUCO/ACCDIS^9^	Maule Cohort/Advanced Center for Chronic Diseases	Chile	Factors involved in development of or protection against CVDs, cancer, diabetes	10,000 unselected adults aged 38 to 74 years from the agricultural town of Molina	2014	http://www.accdis.cl
ESMaestras^10^	Estudio de Salud de la Maestras (Teachers´ Health Study)	Mexico	Lifestyle and health factors involved in development of CVDs, cancer and diabetes in women	115,315 female + 2,160 male teachers	2006	http://www.esmaestras.org/
Cronicas^11^	Centro de Excelencia en Enfermedades Cronicas, Universidad Cayetano Heredia	Peru	Role of geographical and environmental variation as risk factors for chronic disease (CVDs and COPD)	3000 individuals, 35 years of age or older, from 4 regions in Peru: Tumbes, Puno (urban), Puno (rural), and Lima	2010	http://www.cronicas-upch.pe/

This meeting marked a big step forward for Latin American and Caribbean investigators working on the epidemiology of noncommunicable diseases, towards permanent cooperation. In the near future, this should include more studies in those nations and in other countries. The consequences of this aggregation will allow Latin American and Caribbean epidemiological scientists to carve out a role as significant players worldwide, in issues relating to noncommunicable diseases.
